# Supplementary material for: European and multi-ancestry genome-wide association meta-analysis of atopic dermatitis highlights importance of systemic immune regulation
Source: Nat Commun. 2023 Oct 4;14:6172. doi: 10.1038/s41467-023-41180-2 (PMC10550990; doi:10.1038/s41467-023-41180-2)
Supplement: Supplementary file 3 — Description of Additional Supplementary Files [file 41467_2023_41180_MOESM3_ESM.pdf]

Description of Additional Supplementary Files

File Name: Supplementary Data 1
Description: Description: Previously associated variants. Association estimates are derived from the European discovery meta-analysis and the multi-ancestry meta-analysis of the current study. References are listed in the main text. The ancestries within which the associations were originally identified are reported.

File Name: Supplementary Data 2
Description: Cohort details. Characteristics of the European discovery cohorts, multi-ancestry cohorts, and replication cohorts.

File Name: Supplementary Data 3
Description: Conditional analyses. Crude association is the association of the secondary SNP unadjusted for the primary SNP (two-sided test). Conditional association is the association of the secondary SNP adjusted for the primary SNP.

File Name: Supplementary Data 4
Description: Multi-ancestry results. 14 loci meeting genome-wide significance in the multi-ancestry MR-MEGA meta-analysis. Results from a multi-ancestry fixed effects meta-analysis (unadjusted two-sided P-values) are also shown including OR and 95% CI, along with the heterogeneity Q-statistic P-value. Replication estimates are derived from the RIKEN cohort for Japanese individuals, and 23andMe for individuals of Latino, African and European ancestry.

File Name: Supplementary Data 5
Description: Allele frequency comparison. Allele frequencies for the 81 variants from the European discovery meta-analysis, and the 14 variants from the multi-ancestry meta-analysis. Allele frequencies derived from the European discovery meta-analysis, RIKEN cohort and 23andMe for individuals of European, Japanese, Latino and African ancestry respectively.

File Name: Supplementary Data 6
Description: Genetic correlations. Results from LD score regression analysis against all phenotypes available on CTG-VL (5th Nov 2021). Genetic correlations (rg) with the corresponding two-sided p-values (p) are displayed for each tested trait with atopic dermatitis.

File Name: Supplementary Data 7
Description: Garfield DHS peak enrichment. Enrichment was tested at two GWAS P-value threshold (1x10-8 and 1x10-5, PThresh). The OR and two-sided P-value for enrichment are given in the corresponding columns for each cell type tested (only DHS 'peaks' are reported in the main text).

File Name: Supplementary Data 8
Description: MAGMA GTEx gene expression enrichment. Beta, SE and two-sided P for gene expression enrichment are reported for each tissue type tested in the GTEx ver.8 dataset.

File Name: Supplementary Data 9
Description: DEPICT pathway analysis. The nominal two-sided P-value for enrichment is reported for each gene set term tested. Gene sets significant at 5% FDR are highlighted.

File Name: Supplementary Data 10
Description: Post-GWAS gene prioritisation for loci discovered in European meta-analysis – top 3 genes per locus. For each locus, the top 3 genes (along with their Total Evidence Scores) are presented.

File Name: Supplementary Data 11
Description: Post-GWAS gene prioritisation – all genes tested. For each locus, the available evidence implicating each gene tested is summarised. Columns H to U summarises the evidence from specific methods. The final 2 columns display the 2 summary scores generated and used to prioritise genes at each locus.

File Name: Supplementary Data 12
Description: STRING network analysis. Top prioritised genes from the 81 European loci are used as input.

File Name: Supplementary Data 13
Description: eQTL datasets used in post-GWAS prioritisation. Reference numbers correspond to references in the main text.
