# Supplementary material for: European and multi-ancestry genome-wide association meta-analysis of atopic dermatitis highlights importance of systemic immune regulation
Source: Nat Commun. 2023 Oct 4;14:6172. doi: 10.1038/s41467-023-41180-2 (PMC10550990; doi:10.1038/s41467-023-41180-2)
Supplement: Supplementary file 5 — Reporting Summary [file 41467_2023_41180_MOESM5_ESM.pdf]

nature portfolio

Corresponding author(s): Lavinia Paternoster

Last updated by author(s): May 15, 2023

Reporting Summary

Nature Portfolio wishes to improve the reproducibility of the work that we publish. This form provides structure for consistency and transparency in reporting. For further information on Nature Portfolio policies, see our Editorial Policies and the Editorial Policy Checklist.

Statistics

For all statistical analyses, confirm that the following items are present in the figure legend, table legend, main text, or Methods section.

n/a	Confirmed	
☐	☒	The exact sample size (*n*) for each experimental group/condition, given as a discrete number and unit of measurement
☒	☐	A statement on whether measurements were taken from distinct samples or whether the same sample was measured repeatedly
☐	☒	The statistical test(s) used AND whether they are one- or two-sided<br>*Only common tests should be described solely by name; describe more complex techniques in the Methods section.*
☐	☒	A description of all covariates tested
☐	☒	A description of any assumptions or corrections, such as tests of normality and adjustment for multiple comparisons
☐	☒	A full description of the statistical parameters including central tendency (e.g. means) or other basic estimates (e.g. regression coefficient) AND variation (e.g. standard deviation) or associated estimates of uncertainty (e.g. confidence intervals)
☐	☒	For null hypothesis testing, the test statistic (e.g. *F*, *t*, *r*) with confidence intervals, effect sizes, degrees of freedom and *P* value noted<br>*Give P values as exact values whenever suitable.*
☒	☐	For Bayesian analysis, information on the choice of priors and Markov chain Monte Carlo settings
☒	☐	For hierarchical and complex designs, identification of the appropriate level for tests and full reporting of outcomes
☒	☐	Estimates of effect sizes (e.g. Cohen's *d*, Pearson's *r*), indicating how they were calculated

Our web collection on statistics for biologists contains articles on many of the points above.

Software and code

Policy information about availability of computer code

Data collection	This has been detailed in the supplementary methods for each cohort.
Data analysis	For the individual GWAs analysis performed by each cohort, this has been detailed in the supplementary methods. Genetic data was imputed separately for each cohort with either the Michigan or Sanger server. The meta-analysis with European cohorts was performed with GWAMA version 2.2.2. The meta-analysis was performed across all cohorts with MR-MEGA version 0.2. Clumping was performed in PLINK 1.90. GCTA (version 1.92) was used to perform the conditional analysis. Linkage disequilibrium score (LDSC) regression software (version 1.0.1) was used to estimate the SNP-based heritability. Enrichment of tissues and cell types and gene sets was investigated using DEPICT, GARFIELD and MAGMA v.1.06 (using GTEx ver. on the FUMA platform) and MendelVar. Candidate genes were prioritised using eQTL and pQTL data from the eQTL catalogue and Open GWAS. TWAS (Transcriptome-Wide association Study)-based S-MultiXcan and SMR (Summary-based Mendelian Randomization) were run on datasets available via the CTG-VL platform. We also used machine learning candidate gene prioritization pipelines – DEPICT, PoPs, POSTGAP and Open Targets Genetics Variant 2 Gene mapping tool as well as gene-based MAGMA test. VEP (variant Effect Predictor) was used to annotate genes. Network analysis was carried out in STRING v11.5.

For manuscripts utilizing custom algorithms or software that are central to the research but not yet described in published literature, software must be made available to editors and reviewers. We strongly encourage code deposition in a community repository (e.g. GitHub). See the Nature Portfolio guidelines for submitting code & software for further information.

Data

Policy information about availability of data

All manuscripts must include a data availability statement. This statement should provide the following information, where applicable:

- Accession codes, unique identifiers, or web links for publicly available datasets
- A description of any restrictions on data availability
- For clinical datasets or third party data, please ensure that the statement adheres to our policy

Data availability
Summary statistics of the GWAS meta-analyses generated in this study have been deposited in the GWAS Catalog (https://www.ebi.ac.uk/gwas/home) under study accession IDs GCST90244787 and GCST90244788.
The variant-level data for the 23andMe replication dataset are fully disclosed in the main tables and supplementary tables. Individual-level data are not publicly available due to participant confidentiality, and in accordance with the IRB-approved protocol under which the study was conducted.

Human research participants

Policy information about studies involving human research participants and Sex and Gender in Research.

Reporting on sex and gender	All analyses were adjusted for sex. This has been described in the methods section: "GWAS was performed separately for each cohort while adjusting for sex [...]."
Population characteristics	The discovery European meta-analysis included 864,982 participants, 60,653 atopic dermatitis cases and 804,329 controls from 40 cohorts, 25 children and 15 adult cohorts.<br>The multi-ancestry analysis included 1,086,394 individuals (65,107 cases and 1,021,287 controls) from European, Japanese, Latino and African ancestry.<br>Replication included 3,604,027 participants from 23andMe of European, African and Latino ancestry.<br>Definitions of atopic dermatitis are cohort specific, including definitions based on self-report, self-report of doctor-diagnosis or doctor-diagnosis.<br>This has been detailed in the supplementary methods for each cohort.
Recruitment	This has been detailed in the supplementary methods for each cohort. Atopic dermatitis cases were either defined as individuals who has been diagnosed or those who self-reported to be sufferers.
Ethics oversight	This has been detailed in the supplementary methods for each cohort.

Note that full information on the approval of the study protocol must also be provided in the manuscript.

Field-specific reporting

Please select the one below that is the best fit for your research. If you are not sure, read the appropriate sections before making your selection.

☒ Life sciences ☐ Behavioural & social sciences ☐ Ecological, evolutionary & environmental sciences

For a reference copy of the document with all sections, see nature.com/documents/nr-reporting-summary-flat.pdf

Life sciences study design

All studies must disclose on these points even when the disclosure is negative.

Sample size	60,653 atopic dermatitis cases and 804,329 controls were included in the European ancestry meta-analysis, while 65,107 atopic dermatitis cases and 1,021,287 controls were included in the multi-ancestry meta-analysis.
Data exclusions	Genetic variants were restricted to a MAF >1% and an imputation quality score > 0.5 unless otherwise specified in the Supplementary Methods. In order to robustly incorporate cohorts with small sample sizes, we applied additional filtering based on the expected minor allele count (EMAC). EMAC combines information on sample size, MAF and imputation quality (2*N*MAF*imputation quality score) and a threshold of >50 EMAC was used to include variants for all cohorts. QQ-plots and Manhattan plots for each cohort were generated and visually inspected as part of the quality control process.
Replication	The genome-wide index SNPs identified from the European and mixed-ancestry discovery meta-analyses were taken forward for replication in 23andMe, Inc. Individuals of European (N=2,904,664), Latino (N=525,348) and African ancestry (N=174,015) were analysed separately. Full details are available in the Supplementary Methods.
Randomization	Cases were defined as those who have “ever had atopic dermatitis”, according to the best definition for the cohort, where doctor-diagnosed cases were preferred. Controls were defined as those who had never had AD. Further details on the phenotype definitions for the included studies can be found in the Supplementary Methods and Supplementary Table 2

Blinding	Blinding was not relevant as this was a purely observational study and no clinical trail.

Reporting for specific materials, systems and methods

We require information from authors about some types of materials, experimental systems and methods used in many studies. Here, indicate whether each material, system or method listed is relevant to your study. If you are not sure if a list item applies to your research, read the appropriate section before selecting a response.

Materials & experimental systems

n/a	Involved in the study	
☒	☐	Antibodies
☒	☐	Eukaryotic cell lines
☒	☐	Palaeontology and archaeology
☒	☐	Animals and other organisms
☐	☒	Clinical data
☒	☐	Dual use research of concern

Methods

n/a	Involved in the study	
☒	☐	ChIP-seq
☒	☐	Flow cytometry
☒	☐	MRI-based neuroimaging

Clinical data

Policy information about clinical studies

All manuscripts should comply with the ICMJE guidelines for publication of clinical research and a completed CONSORT checklist must be included with all submissions.

Clinical trial registration	Not applicable
Study protocol	Not applicable
Data collection	This has been detailed in the supplementary methods for each cohort.
Outcomes	Atopic Dermatitis
